# Propylthiouracil-induced ANCA-associated vasculitis complicated by granulocytopenia and hemophagocytosis: a case report

**DOI:** 10.3389/fmed.2025.1578726

**Published:** 2025-04-09

**Authors:** Huilin Zhou, Shuchang Lai, Jinyi Chen, Yi Wang, Shasha Fu, Zongcun Chen

**Affiliations:** ^1^Department of Endocrinology, The Second Affiliated Hospital of Hainan Medical University, Hainan, China; ^2^Department of Respiratory and Critical Care Medicine, Haikou Affiliated Hospital of Central South University Xiangya School of Medicine (Haikou People’s Hospital), Hainan, China

**Keywords:** ANCA-associated vasculitis, propylthiouracil, hemophagocytosis, hyperthyroidism, granulocytopenia

## Abstract

**Objective:**

To analyze a rare case of ANCA-associated vasculitis (AAV) complicated by hemophagocytosis and granulocytopenia induced by long-term propylthiouracil (PTU) therapy, providing insights for clinical diagnosis and management.

**Methods:**

A retrospective analysis was conducted on the clinical data and treatment course of a patient who developed AAV with hemophagocytosis and granulocytopenia after prolonged PTU use.

**Results:**

Upon admission, granulocytopenia secondary to PTU was suspected. Despite transient recovery of leukocyte counts with anti-infective therapy and granulocyte colony-stimulating factor (G-CSF), recurrent leukopenia and intermittent fever persisted. Bone marrow aspiration revealed hemophagocytic cells, while serologic testing showed positivity for both PR3-ANCA and MPO-ANCA. A definitive diagnosis of PTU-induced AAV was established. Glucocorticoid therapy normalized body temperature and restored leukocyte levels. Follow-up demonstrated resolution of thyrotoxicosis, stabilized leukocyte counts, and afebrile status.

**Conclusion:**

Long-term PTU therapy may trigger AAV accompanied by hemophagocytosis. Clinicians should consider screening for hemophagocytic lymphohistiocytosis (HLH) in such cases to guide timely immunosuppressive intervention.

## Background

Propylthiouracil (PTU) and methimazole (MMI) are frequently employed in the management of hyperthyroidism. These medications predominantly act by suppressing the activity of thyroid peroxidase, thereby curtailing the synthesis of thyroid hormones. Unlike methimazole (MMI), PTU also inhibits the peripheral conversion of thyroxine (T4) to triiodothyronine (T3) (1). The common adverse effects of antithyroid drugs encompass rash, gastrointestinal manifestations, hepatic function derangements, arthralgia, myalgia, leukopenia, and, in severe cases, granulocytopenia (2–4). Antineutrophil cytoplasmic antibody (ANCA) - associated vasculitis represents a relatively infrequent adverse reaction associated with PTU (5, 6). The coexistence of hemophagocytosis in this context is exceptionally rare. Herein, we present a case of ANCA - associated vasculitis, concurrent with hemophagocytosis and granulocytopenia, subsequent to long - term PTU administration. This case offers valuable insights for the clinical diagnosis and treatment of similar patients.

## Case presentation

A 58-year-old man with a decade-long history of hyperthyroidism was admitted on September 19, 2024, complaining of recurrent hand tremors and 5 days of fever. More than ten years prior, the patient experienced the onset of hand tremors, palpitations, and asthenia without an apparent etiology. These symptoms were accompanied by heat intolerance, diaphoresis, and mild exophthalmos, but diplopia was absent. He was diagnosed with “hyperthyroidism” at a local medical facility and had been on an irregular, long-term regimen of “propylthiouracil” (manufactured by Herbrand Pharma Chemicals GmbH, Germany). Three months prior to admission, the patient transitioned to a different brand of PTU, taking 50 mg twice daily, produced by Jinghua Pharmaceutical Group. Five days before admission, the patient developed paroxysmal episodes of cough and dyspnea without an obvious precipitating factor. The cough was non-productive, and these symptoms were accompanied by chest tightness and fever, with the body temperature peaking at 38.6°C.

On admission, vital signs included a temperature of 38°C, heart rate of 115 bpm, respiratory rate of 22 breaths per minute, and a blood pressure of 102/65 mmHg. The patient manifested an acute febrile facies, maintained an autonomous posture, and was fully conscious. Bilateral eyeballs exhibited no remarkable exophthalmos. Auscultation of both lungs revealed coarse breath sounds, with no audible dry or wet rales. The heart rate remained at 115 beats per minute, the cardiac rhythm was regular, and no cardiac murmurs were detected upon auscultation. The bilateral thyroid glands were enlarged to grade II, with a medium - consistency texture, were freely movable, without audible vascular murmurs, and no nodules were palpable. When the patient extended both hands horizontally, fine tremors were observable.

**Figure 1:**
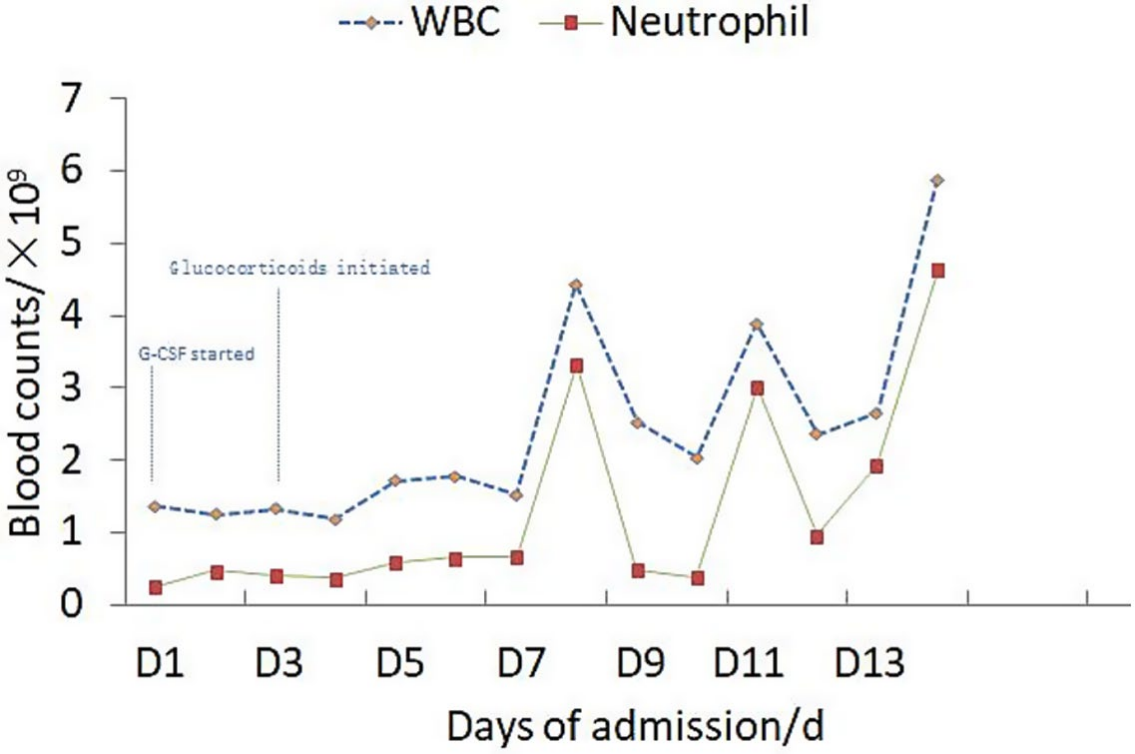
WBC and neutrophil trends with treatment timeline.

**Figure 2:**
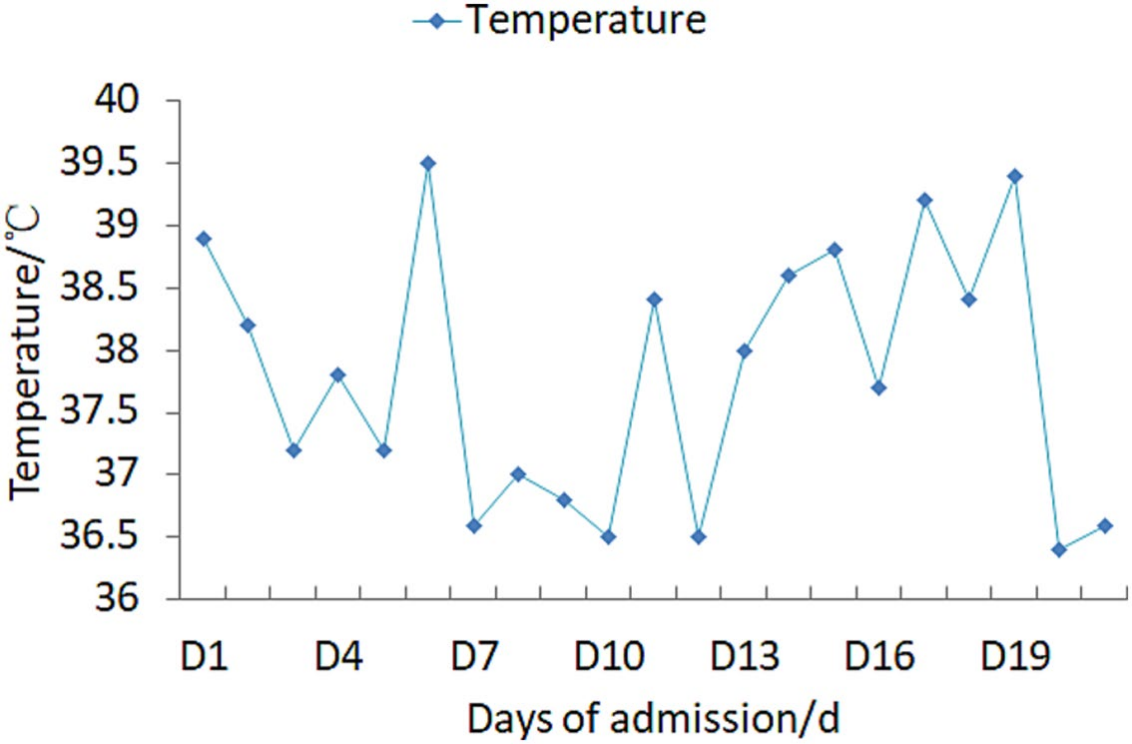
Body temperature profile during treatment.

Upon admission, the patient manifested leukopenia and neutropenia accompanied by fever. To augment the white blood cell count, a regimen comprising human granulocyte - colony stimulating factor injection, Diyu Shengbai Capsules, adenosine phosphate tablets, and batyl alcohol tablets was promptly instituted. Subsequently, the patient developed intermittent fever. Serial hematologic tests demonstrated prolonged pancytopenia. Chest imaging revealed scattered patchy and cord-like opacities bilaterally, raising suspicion of infection. Consequently, empirical antimicrobial therapy was initiated with piperacillin-sulbactam followed by meropenem. Concurrently, blood cultures, influenza A/B viral testing, and bone marrow biopsy were performed. No pathogenic organisms were detected in blood cultures, and viral tests were negative. Despite anti-infective therapy, the patient’s fever persisted. Given suspected immune-mediated pathology, methylprednisolone sodium succinate was initiated for immunosuppression, administered once daily at 40 mg. This treatment was terminated on September 25, 2024. Throughout the treatment course, the white blood cell and neutrophil counts initially exhibited a transient elevation but subsequently underwent a rapid decline (Figure 1), concomitantly with persistent intermittent fever, with the body temperature peaking at 39.5°C.On September 29, 2024, the bone marrow aspiration findings revealed active proliferation of all three hematopoietic lineages, with discernible hemophagocytic histiocytes (Figure 2). Given the patient’s protracted fever (body temperature > 38.5°C), pancytopenia, and the abdominal computed tomography (CT) - detected splenomegaly, the possibility of hemophagocytic lymphohistiocytosis could not be discounted. A re - assessment of the complete blood count on October 2, 2024, disclosed a white blood cell count of 2.36 × 10^9^/L, signifying a recurrence of leukopenia. Consequently, the therapeutic interventions aimed at leukocytosis promotion and anti - infection were continued. Owing to the indeterminate etiology of the patient’s long - standing fever, leukopenia, and neutropenia, additional diagnostic investigations were pursued. The results of anti - blood cell antibody (quantitative) assays were as follows: Urinalysis: Urine occult blood (2+) was detected, anti - proteinase 3 antibody PR3 - IgG measured 400.00 AU/mL, and anti - myeloperoxidase antibody MPO - IgG was 286.30 AU/mL. The ferritin level was determined to be 813 ng/mL (reference range: 24–425 ng/mL). The results of all laboratory data are shown in Tables 1, 2.

**Table 1:** Laboratory data.

Examination items	Test results	Normal range
White blood cells (x10^9^/L)	1.36	3.5–9.5
Neutrophils (x10^9^/L)	0.24	1.8–6.3
Red blood cells (x10^12^/L)	3.91	4.3–5.8
Platelets (x10^9^/L)	103	100–300
Hemoglobin (g/L)	109	130–175
Interleukin – 6 (pg/mL)	128	0–7
C - Reactive Protein (mg/L)	110.4	0–10
Procalcitonin (ng/L)	0.99	0–0.5
Erythrocyte sedimentation rate (mm/L)	61	0–15
Triiodothyronine (ng/mL)	2.53	0.75–2.1
Total Thyroxine (ng/mL)	148	50–130
Free Triiodothyronine (pg/mL)	4.39	2–4.2
Free Thyroxine (pg/mL)	29.60	9–17.5
Thyroid - Stimulating hormone (μIU/mL)	<0.001	0.3–4.5
Anti - thyroid peroxidase antibody (IU/mL)	18.60	0–10
Anti - thyroglobulin antibody (IU/mL)	2004.00	0–95
Thyroglobulin (ng/mL)	<0.02	3.5–77
Thyrotropin receptor antibody (IU/L)	26.40	0–1.5
Aspartate transaminase (U/L)	21	15–40
Alanine transaminase (U/L)	32	9–50
serum creatinine (μmol/L)	66	57–97
blood urea nitrogen (mmol/L)	4.35	2.9–8.0
Triglycerides (mmol/L)	1.52	0.56–1.70
Lactate dehydrogenase (U/L)	215	135–225
Aspartate transaminase(U/L)	21	15–40
Alanine transaminase (U/L)	32	9–50
Serum creatinine (μmol/L)	66	57–97
Blood urea nitrogen (mmol/L)	4.35	2.9–8.0
Triglycerides (mmol/L)	1.52	0.56–1.70
Lactate dehydrogenase(U/L)	215	135–225
Fibrinogen (g/L)	5.06	2–4
D – dimer (μg/mL)	1.22	0–0.55
Ferritin (ng/mL)	813	24–425

**Table 2:** Immunological and serological tests.

Examination items	Test results	Normal range
Anti - glomerular basement membrane antibody GBM – IgG(AU/ml)	2.00	0–24
Anti - proteinase 3 antibody PR3 – IgG(AU/ml)	400.00	0–24
Anti - myeloperoxidase antibody MPO – IgG(AU/ml)	286.30	0–24
Antinuclear antibody	Negative	
Antinuclear antibody extract	Negative	
Rheumatoid factor	Negative	
Immunoglobulin G content determination (g/L)	20.27	8.6–17.4
Immunoglobulin M content determination (g/L)	2.499	0.3–2.2
κ Light chain (g/L)	5.250	1.38–3.75
λ Light chain (g/L)	2.650	0.93–2.42
Gastrin 17(pmol/L)	19.2	1.7–7.6
Carbohydrate antigen 125 (U/mL)	46.3	0–35
Pepsinogen I	Negative	
Pepsinogen II	Negative	
PGI/PGII	Negative	
Prostate Tumor Markers	Negative	
COVID - 19 nucleic acid test	Negative	
Influenza A - IGM	Negative	
Influenza B - IGM	Negative	
Fungal (1–3)-β - D glucan detection (pg/mL)	278.03	0–100
Respiratory pathogen joint detection	Negative	
TORCH	Negative	
*Mycobacterium tuberculosis* gamma - interferon Release assay	Negative	
Blood culture	Negative	
Sputum culture	Negative	
Widal test	Negative	
Weil - felix reaction	Negative	
Plasmodium	Negative	

**Figure 3:**
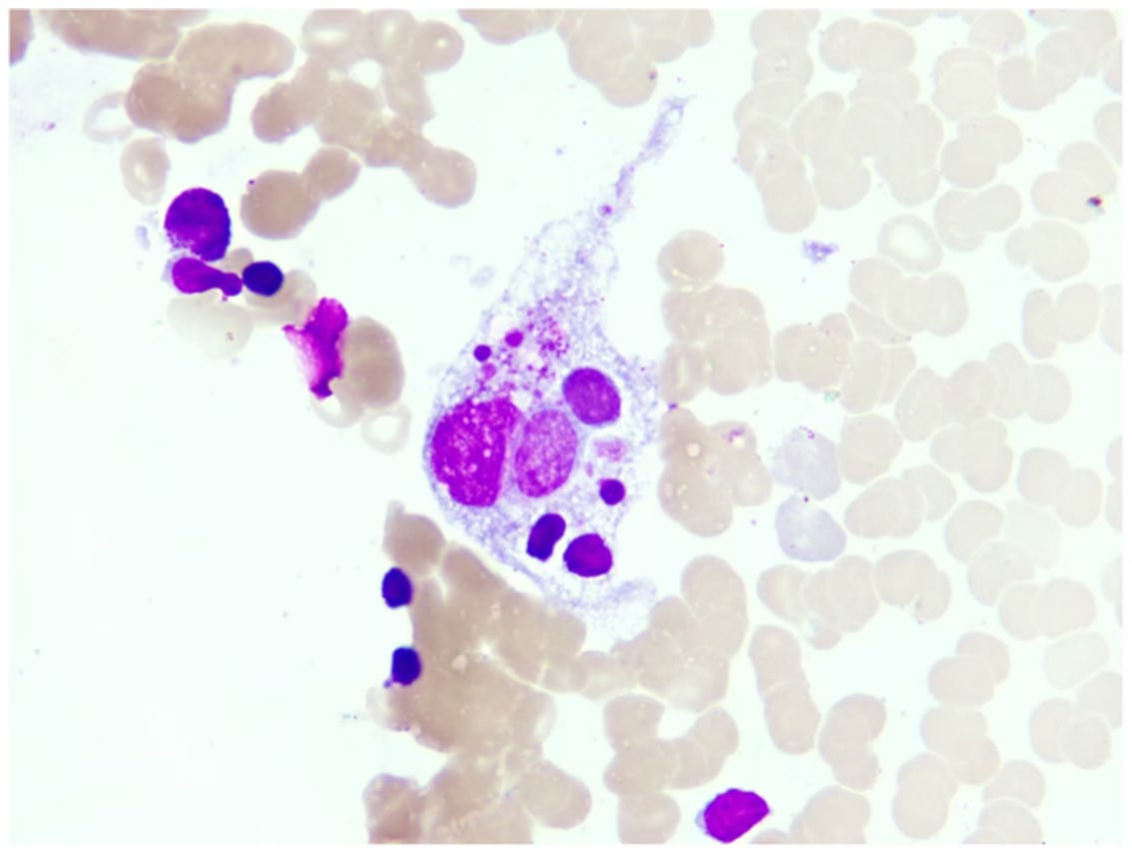
This is the macrophage phagocytosis phenomenon observed under the microscope in the patient’s bone marrow image (magnification 1,000X).

The administration of propylthiouracil (PTU) to the patient was promptly discontinued. Subsequently, a sequential anti - infectious regimen was initiated, commencing with meropenem followed by piperacillin - sulbactam. Concurrently, pharmacological interventions aimed at augmenting the white blood cell count were implemented, including the use of human granulocyte - colony stimulating factor (G - CSF) injection, Diyu Shengbai Capsules, adenosine phosphate tablets, and batyl alcohol tablets. Intravenous methylprednisolone sodium succinate (40 mg daily) was initiated as anti-inflammatory therapy. Throughout the treatment period, the outcomes of the leukocytosis - promoting therapies, as evidenced by the white blood cell and neutrophil counts, were suboptimal (refer to Figure 1 for detailed data).After approximately two weeks, the antibiotic therapy was terminated, while the administration of methylprednisolone sodium succinate was continued. Twenty days following this adjustment, the patient’s body temperature normalized (Figure 3). A subsequent complete blood count demonstrated the restoration of the white blood cell count to within the normal range, signifying an improvement in the patient’s clinical condition. Subsequently, the patient opted for radioactive iodine - 131 treatment. Post - discharge, the patient continued with an oral prednisone tablet regimen. One-month follow-up assessment revealed normalization of body temperature, leukocyte count, neutrophil count, and erythrocyte sedimentation rate. Notably, the urine occult blood test turned negative. Thyroid function reassessment showed elevated free thyroxine (FT4) at 20 pg./mL (reference range: 9–17.5 pg./mL), free triiodothyronine (FT3) within the upper limit of normal (4.16 pg./mL; reference: 2–4.2 pg./mL), and markedly suppressed thyroid-stimulating hormone (TSH) at 0.002 μIU/mL (reference: 0.3–4.5 μIU/mL). At the 4-month follow-up, ANCA serology dynamics demonstrated persistent elevation of anti-proteinase 3 antibody (PR3-IgG: 400 AU/mL) alongside a decline in anti-myeloperoxidase antibody (MPO-IgG: 227.80 AU/mL).

## Discussion

In the context of this hyperthyroidism case, subsequent to the substitution of Propylthiouracils (PTUs) from different manufacturers, manifestations such as fever, granulocyte deficiency, and ANCA - associated vasculitis with hemophagocytosis emerged. Reports of similar cases remain scarce, and there persist several contentious issues within the diagnostic and therapeutic processes.

Upon admission, the patient was found to have leukopenia and agranulocytosis (granulocyte count <0.5 × 10^9^/L). Agranulocytosis is one of the severe adverse drug reactions of PTU. The occurrence of agranulocytosis is related to the type, dose and application time of antithyroid drugs. Yoshimura et al. (7) discovered that both Methimazole (MMI) and Propylthiouracil (PTU), two medications used in the treatment of thyroid - related conditions, exhibited a dose - dependent elevation in the incidence of granulocyte deficiency. Specifically, when considering equipotent dosages in terms of thyroid hormone synthesis inhibition, PTU was found to have a significantly higher propensity to induce granulocyte deficiency compared to MMI. Granulocytopenia characteristically manifests within the initial three - month period subsequent to the commencement of antithyroid drug (ATD) therapy (8). Nevertheless, instances have been documented wherein the onset transpired following an exposure duration exceeding 10 years (9). The underlying pathogenesis of propylthiouracil (PTU)-induced agranulocytosis remains incompletely elucidated. It is potentially associated with the inhibition of nucleic acid metabolism in bone marrow granulocytes. PTU has the ability to trigger the generation of anti - neutrophil cytoplasmic antibodies (ANCA) within the body, thereby inciting an autoimmune reaction. Once neutrophils are sensitized and the antigens translocate to the cell membrane, ANCA can bind to neutrophil antigens, including protease - 3 (PR3), myeloperoxidase (MPO), and cathepsin G, which leads to neutrophil degranulation (10).In the present case, granulocyte colony - stimulating factor was administered to elevate the white blood cell count, meropenem, piperacillin sodium, and sulbactam sodium were utilized for anti - infection purposes, and hormones were applied for a short - term. However, the white blood cell count increased transiently and then decreased again, indicating suboptimal treatment efficacy. PTU - induced agranulocytosis typically manifests 3–6 months after the initiation of drug use, and it can also occur during long - term administration. Nevertheless, in most cases, the white blood cell count can be restored to normal levels following leukocyte - elevating treatment. In this particular patient, the poor response to leukocyte - elevating treatment is presumably related to other diseases that cause leukopenia, such as autoimmune disorders and hematological malignancies.

The patient had no antecedent medical history of renal or pulmonary disorders and exhibited symptoms such as fever, weight loss, and cough. The urine occult blood test demonstrated a positive outcome, and the pulmonary imaging depicted scattered patchy and cord-like regions of elevated density in both lungs. Both PR3-IgG and MPO-IgG were strongly positive. In light of the history of Propylthiouracil (PTU) administration, ANCA-associated vasculitis induced by PTU was suspected. AAV encompasses a spectrum of autoimmune small-vessel vasculitides characterized histologically by fibrinoid necrosis and serologically by circulating ANCAs targeting neutrophil cytoplasmic antigens. This encompasses granulomatosis with polyangiitis (GPA), microscopic polyangiitis (MPA), and eosinophilic granulomatosis with polyangiitis (EGPA). BALAVOINE et al. conducted a comprehensive review of the case reports of 260 patients afflicted with antithyroid drug-related ANCA-associated vasculitis (AAV). It was discovered that 75% of these cases were correlated with propylthiouracil, while 25% were associated with methimazole. Juvenile age and extended treatment duration constitute the primary risk factors for ANCA positivity (11). There exists an interaction between Propylthiouracil (PTU) and ANCA-targeted antigens (primarily proteinase 3 (PR3) and myeloperoxidase (MPO)). PTU is capable of inducing the generation of ANCA, and ANCA further facilitates the over-activation of neutrophils, which subsequently release inflammatory cytokines, reactive oxygen species, and proteases, thereby inflicting damage on vascular endothelial cells and resulting in AAV (12). Furthermore, the hyperactivation of neutrophils mediated by ANCA has the potential to induce the formation of neutrophil extracellular traps (NETs). NETs exert a cytotoxic influence on vascular endothelial cells and are mainly degraded by deoxyribonuclease I (DNase I) in the serum. Propylthiouracil (PTU) is able to inhibit the activity of DNase I. As a result, this inhibition gives rise to the accumulation of NETs in the body, which further impairs vascular endothelial cells, eventually leading to the development of ANCA - associated vasculitis (AAV) (13). Indirect immunofluorescence assay can identify two distinct subtypes of ANCA: cytoplasmic ANCA (cANCA) and perinuclear ANCA (pANCA). For cANCA, the principal target antigen is proteinase 3 (PR3), while for pANCA, it is predominantly myeloperoxidase (MPO). In cases of Propylthiouracil (PTU)-induced ANCA - associated vasculitis (AAV), a double - positive profile is frequently observed, signifying the concurrent detection of both PR3 - ANCA and MPO - ANCA (14).

The clinical presentations of drug - induced ANCA - associated vasculitis bear resemblance to those of primary vasculitis. Virtually any organ system may be affected. Oftentimes, the inaugural symptoms manifest as fever or cutaneous manifestations, with the lungs and kidneys being the most commonly implicated organs (13). In cases where the pulmonary system is affected, common clinical manifestations typically encompass cough and dyspnea. In severe instances, this condition may progress to pulmonary hemorrhage. From a radiological perspective, findings commonly include patchy, mottled, or linear opacities with increased density; alternatively, extensive areas of consolidation or ground - glass opacities may be observed. When renal involvement occurs, it is usually characterized by hematuria and proteinuria. Regarding therapeutic management, the immediate cessation of Propylthiouracil (PTU) is imperative. In certain patients, symptom alleviation may ensue following the discontinuation of PTU. Nevertheless, for individuals presenting with vital organ involvement, immunosuppressive therapy, encompassing the administration of corticosteroids and immunosuppressants, becomes requisite. In cases of refractory ANCA - associated vasculitis (AAV), plasmapheresis and biological agents may be employed (15). The patient presented in this case had an extensive history of Propylthiouracil (PTU) intake with concomitant involvement of both the pulmonary and renal systems. A dual - positive status for Proteinase 3 - Anti - Neutrophil Cytoplasmic Antibody (PR3 - ANCA) and Myeloperoxidase - Anti - Neutrophil Cytoplasmic Antibody (MPO-ANCA) was detected. Following a comprehensive assessment, the patient’s clinical condition demonstrated improvement subsequent to the administration of corticosteroid pulse therapy.In patients with a protracted course of PTU utilization who develop leukopenia and exhibit an inadequate response to leukocytosis - promoting interventions, the potential presence of ANCA - associated vasculitis should be thoroughly evaluated.

The results of the patient’s bone marrow aspiration revealed the presence of hemophagocytic histiocytes. Clinicians should be vigilant about the potential development of hemophagocytic lymphohistiocytosis (HLH). Hemophagocytic lymphohistiocytosis (HLH) represents a syndrome characterized by an excessive inflammatory response. From a clinical perspective, it typically presents with fever, pancytopenia, hepatosplenomegaly, and the identification of activated macrophages within hematopoietic organs. Prognostically, HLH generally portends a poor outcome, and in severe instances, it may culminate in death. HLH can be subclassified into primary and secondary forms. Primary HLH, more frequently encountered in pediatric patients, is an autosomal or X - linked recessive genetic condition. In contrast, secondary HLH is more commonly observed in adult populations and may arise secondary to a diverse array of etiologies, including infections caused by viruses, bacteria, and parasites, as well as rheumatologic and immunological disorders, metabolic derangements, and neoplasms.

At present, the HLH - 2004 criteria formulated by the Histiocyte Society are widely adopted internationally as the diagnostic standards for hemophagocytic lymphohistiocytosis (HLH). Specifically, a diagnosis of HLH can be established if either of the following two conditions is satisfied:

1. Identification of molecular - genetic abnormalities associated with HLH;
2. Meeting 5 out of the following 8 diagnostic criteria:

(1) Persistent fever for more than 7 days;
(2) Enlargement of the spleen (splenomegaly);
(3) Cytopenia affecting two or three hematopoietic lineages: Hemoglobin level below 90 g/L (in infants younger than 4 weeks, below 100 g/L), platelet count less than 100 × 10^9^/L, and neutrophil count lower than 1.0 × 10^9^/L;
(4) The presence of both hypertriglyceridemia and/or hypofibrinogenemia, characterized by fasting triglyceride levels exceeding 3.0 mmol/L and fibrinogen levels below 1.5 g/L;
(5) Visualization of hemophagocytosis in bone - marrow aspirates, splenic tissue, or lymph - node specimens;
(6) Diminished or absent natural killer (NK) cell activity;
(7) Serum ferritin levels greater than 500 μg/L;
(8) Elevated plasma levels of soluble CD25 (SIL - 2R) above 2,400 U/mL or an increased lactate dehydrogenase level.

In the present case, the patient fulfilled five criteria within the second category, specifically including fever, splenomegaly, pancytopenia, the detection of hemophagocytosis in bone marrow specimens, and a ferritin level exceeding 500 μg/L. Consequently, the diagnostic benchmarks for hemophagocytic lymphohistiocytosis (HLH) were met. Nonetheless, contemporary research posits that hemophagocytosis is no longer regarded as a requisite and conclusive condition for the diagnosis of HLH (16, 17). Additionally, hemophagocytosis can manifest in autoimmune disorders. In comparison to hemophagocytosis, aberrant liver function, ferritin concentrations, and natural killer (NK) cell activity carry greater diagnostic significance for HLH (16). The HLH - 2004 diagnostic guidelines specify a serum ferritin level surpassing 500 ng/mL. Nevertheless, investigations have indicated that a serum ferritin level exceeding 2000 ng/mL is commonly regarded as raising suspicion for HLH, and a level exceeding 10,000 ng/mL substantially enhances the diagnostic sensitivity and specificity for HLH, particularly in adult patients (17, 18).

The patient exhibited normal liver and kidney functions as well as triglyceride levels, with an insignificant elevation in ferritin. Taking into account the clinical presentations of fever and pancytopenia, in conjunction with the patient’s pulmonary imaging findings, a stronger association with ANCA - associated vasculitis was deemed more probable. As a result, the diagnosis of hemophagocytic lymphohistiocytosis was temporarily excluded. The subsequent follow - up outcomes of the patient further precluded the possibility of HLH. In individuals afflicted with autoimmune diseases, autoantibodies may play a mediatory role, and circulating immune complexes can deposit on bone - marrow hematopoietic cells. This deposition augments the susceptibility of phagocytes, potentially culminating in the onset of hemophagocytic lymphohistiocytosis (HLH). A retrospective analysis encompassing the medical records, diagnostic algorithms, and therapeutic trajectories of 55 patients definitively diagnosed with autoimmune - disease - associated hemophagocytic syndrome (AAHS) reveals that a diverse spectrum of autoimmune disorders possess the propensity to precipitate hemophagocytic syndrome (HPS) (19). In the present case, the hemophagocytosis detected in the patient’s bone - marrow specimens cannot be excluded as being attributable to ANCA - associated vasculitis. Nevertheless, to date, there have been no documented reports of HLH precipitated by ANCA - associated vasculitis.

Autoimmune disorders have the potential to induce hemophagocytosis. In the absence of early recognition, this phenomenon may advance to hemophagocytic lymphohistiocytosis (HLH). When patients diagnosed with autoimmune diseases exhibit manifestations such as high - grade fever, cytopenia, splenomegaly, and impairment of hepatic and renal functions, the likelihood of HLH should be taken into account. HLH is a life-threatening hyperinflammatory syndrome characterized by cytokine storm, which may complicate diverse inflammatory conditions. Characterized by a rapid progression, early identification and appropriate management of HLH are of paramount importance in averting organ failure and mortality (18).

In conclusion, we present a case of a patient with hyperthyroidism who developed agranulocytosis concurrent with ANCA - associated vasculitis and hemophagocytosis subsequent to long - term administration of propylthiouracil (PTU). Presently, the body of literature regarding such cases remains limited.

This case serves as a reminder that when patients exhibit leukopenia, particularly pancytopenia, subsequent to PTU intake (encompassing both prolonged use and switching between different pharmaceutical manufacturers), and the therapeutic interventions aimed at leukocytosis promotion and anti - infection prophylaxis yield suboptimal results, it is imperative to promptly conduct comprehensive evaluations for hematological disorders, connective tissue diseases, and autoimmune pathologies to elucidate the underlying etiology.ANCA - associated vasculitis and hemophagocytic lymphohistiocytosis (HLH) manifest overlapping clinical features, including fever, leukopenia, and involvement of the integumentary and mucosal surfaces. Hence, meticulous differentiation between these two entities is essential. Moreover, hemophagocytosis can also be observed in the context of autoimmune diseases. Therefore, during the diagnostic work - up of ANCA - associated vasculitis, screening for HLH should be incorporated into the assessment protocol.

## Data Availability

The original contributions presented in the study are included in the article/supplementary material. Further inquiries can be directed to the corresponding authors.
